# P-1454. Characterizing the Prevalence of Recurrent Antimicrobial Resistant Infections in an Urban County

**DOI:** 10.1093/ofid/ofae631.1626

**Published:** 2025-01-29

**Authors:** Tanvi A Ingle, Alaina M Beauchamp, Lauren N Cooper, Abdi Wakene, Marlon I Diaz, John J Hanna, Christoph U Lehmann, Richard J Medford

**Affiliations:** University of Texas Southwestern Medical Center, Dallas, Texas; University of Texas Southwestern Medical Center, Dallas, Texas; University of Texas Southwestern Medical Center, Dallas, Texas; University of Texas Southwestern Medical Center, Dallas, Texas; Texas Tech University Health Sciences Center, El Paso, Texas; University of Texas Southwestern, Dallas, Texas; UT Southwestern, Dallas, Texas; ECU Health, Greenville, North Carolina

## Abstract

**Background:**

Individuals treated for antimicrobial resistant organism (AMRO) infections are exposed to broader antibiotics, which can contribute to an increased likelihood of future resistant infections. Given that community transmission and AMRO recurrence remains underexplored, we conducted spatiotemporal analyses of recurrent AMRO infections in an urban county.

Figure 1

**Figure:**
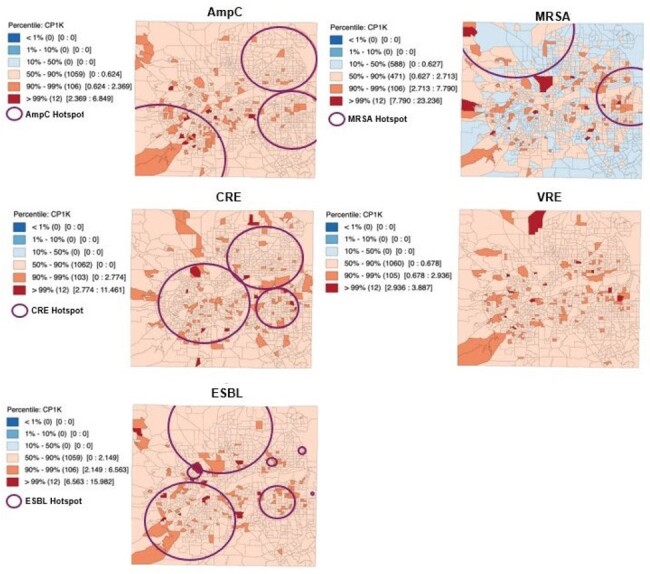

Map of spatiotemporal hotspots and recurrent infection counts per 1,000 population (CP1K) for five patterns of antimicrobial resistance at a census block group level.

**Methods:**

We mapped the spatial prevalence of recurrent AMRO infections for census block groups using electronic health records from two major health systems in Tarrant County (Texas) from 2010-2019. We delineated recurrence across five different patterns: AmpC beta-lactamase producing bacteria (AmpC), Carbapenem-Resistant *Enterobacterales* (CRE), *Extended spectrum beta lactamase* (ESBL), Methicillin-Resistant *Staphylococcus aureus* (MRSA), and Vancomycin-resistant *Enterococcus* (VRE). We compared recurrent AMRO rates against space-time permutation model identified hotspots. We used spatial lag regressions to explore the relationship between recurrent AMRO rates and Area Deprivation Index (ADI), a measure of neighborhood inequity.

**Results:**

Of 105,291 patients, 19,161 (18.2%) had at least one AMRO infection. There were 10,611 (10.1%) patients with multiple AMRO infections: AmpC = 202, CRE = 136, ESBL = 741, MRSA = 1255, and VRE = 205. We observed that AmpC, ESBL, and MRSA recurrence had the greatest co-location with spatiotemporal hotspots (Figure 1). In spatial regressions, increasing neighborhood ADI rank (increasing deprivation) was significantly associated with an increase in block group-rates of recurrent AmpC (β = 0.02), ESBL (β = 0.03), MRSA (β = 0.04) and VRE (β = 0.01) (p-value < 0.05).

**Conclusion:**

We geographically and temporally identified patterns of recurrent AMROs in a large urban county and demonstrated a significant relationship between inequity and AMRO recurrence. This work can enhance our understanding of local infection dynamics and inform antibiotic stewardship.

**Disclosures:**

**John J. Hanna, MD**, Pieces Technologies: Advisor/Consultant

